# The Generation and Modulation of Distinct Gamma Oscillations with Local, Horizontal, and Feedback Connections in the Primary Visual Cortex: A Model Study on Large-Scale Networks

**DOI:** 10.1155/2021/8874516

**Published:** 2021-01-18

**Authors:** Chuanliang Han, Tian Wang, Yujie Wu, Yang Li, Yi Yang, Liang Li, Yizheng Wang, Dajun Xing

**Affiliations:** ^1^State Key Laboratory of Cognitive Neuroscience and Learning & IDG/McGovern Institute for Brain Research, Beijing Normal University, Beijing 100875, China; ^2^Beijing Institute of Basic Medical Sciences, Beijing 100850, China

## Abstract

Gamma oscillation (GAMMA) in the local field potential (LFP) is a synchronized activity commonly found in many brain regions, and it has been thought as a functional signature of network connectivity in the brain, which plays important roles in information processing. Studies have shown that the response property of GAMMA is related to neural interaction through local recurrent connections (RC), feed-forward (FF), and feedback (FB) connections. However, the relationship between GAMMA and long-range horizontal connections (HC) in the brain remains unclear. Here, we aimed to understand this question in a large-scale network model for the primary visual cortex (V1). We created a computational model composed of multiple excitatory and inhibitory units with biologically plausible connectivity patterns for RC, FF, FB, and HC in V1; then, we quantitated GAMMA in network models at different strength levels of HC and other connection types. Surprisingly, we found that HC and FB, the two types of large-scale connections, play very different roles in generating and modulating GAMMA. While both FB and HC modulate a fast gamma oscillation (around 50-60 Hz) generated by FF and RC, HC generates a new GAMMA oscillating around 30 Hz, whose power and peak frequency can also be modulated by FB. Furthermore, response properties of the two GAMMAs in a network with both HC and FB are different in a way that is highly consistent with a recent experimental finding for distinct GAMMAs in macaque V1. The results suggest that distinct GAMMAs are signatures for neural connections in different spatial scales and they might be related to different functions for information integration. Our study, for the first time, pinpoints the underlying circuits for distinct GAMMAs in a mechanistic model for macaque V1, which might provide a new framework to study multiple gamma oscillations in other cortical regions.

## 1. Introduction

Gamma oscillation in the LFP of the visual cortex is thought to play important roles in synchronizing neurons' response in the local network [1, 2] of many brain regions [3–6]. Many recent studies have been focusing on the cognitive functions [7–9], neural mechanisms [10–15], and neural origins [16–22] of gamma oscillation. However, how neural connectivity patterns affect the property of gamma oscillation in the primary visual cortex of macaque (V1) remains unclear.

Anatomical studies [23–28] have shown that V1 has rich neural connection patterns, including feed-forward connection (FF), local recurrent connection (RC), long-distance horizontal connection (HC), and feedback connection (FB). These connection patterns have distinct characteristics and together they form a complex dynamic system for V1. The local circuit (RC) of V1 is driven by lateral geniculate nucleus (LGN) through FF connections and then sends FF projections to several higher-level extrastriate cortical areas. Within V1, the processing of visual information is accomplished through reciprocal local RC and long-range horizontal connection (HC) between excitatory and inhibitory neurons [20, 29, 30]. HC was thought to be implicated by several perceptual phenomena like “filling in” [31] and “illusory contours” [32, 33]. Besides, FB connections are assumed to serve to modulate the neural response of V1 in a large spatial scale with a retinotopic organization less precise than FF projections have [34–37]. These different connection patterns provide a foundation for V1 diverse functional properties [38, 39] including gamma oscillation [4].

Previous studies have shown that gamma oscillation could be generated in a local RC circuit [10, 19, 40–45]. Furthermore, gamma oscillation in V1 was thought to be modulated by FB connection from higher-level brain regions [4, 46] and exert FF influence of gamma oscillation on these downstream visual areas [5, 6, 40, 47–49]. Although researchers have built models with long-range horizontal connections to understand V1 functions [30, 50–52], the relationship between gamma oscillation and HC was absent in the previous literatures.

In this paper, we aimed to understand the functional role of HC for gamma oscillation in a large-scale network model for the primary visual cortex (V1). We created a computational model composed of multiple excitatory and inhibitory units with biologically plausible connectivity patterns for FF, RC, FB, and HC; then, we quantitated gamma oscillations in network models with different strength levels of connection types (HC and FB). In the end, we compared the relationship between gamma oscillation and different types of neural connectivity.

## 2. Methods

### 2.1. Basic Model with E-I Unit

**Figure 1:**
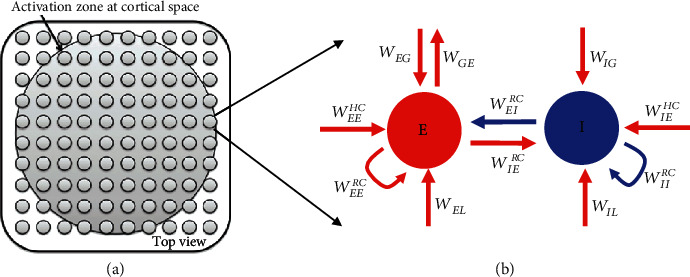
The network architecture of a large scale network model. Small grey circular patches in (a) represent 15 by 15 local components in the network. These local components are placed horizontally on a plane parallel to cortical surface which mimic hypercolumns in V1. The spatial range of activation zone, or the number of activated E-I units in the model, is dependent on the size of external visual stimulus. Each local component in (b) consists of excitatory (E) and inhibitory (I) component connected with local connection *W*_*EE*_^*RC*^, *W*_*EI*_^*RC*^, *W*_*IE*_^*RC*^, and *W*_*II*_^*RC*^. In the cortical space, the E and I components receive feedforward input (*W*_*EL*_, *W*_*IL*_) from subcortical regions (LGN) that are driven by visual stimuli, horizontal inputs (*W*_*EE*_^*HC*^ and *W*_*IE*_^*HC*^) from other E-I units and receives feedback (*W*_*EG*_, *W*_*IG*_) from higher visual cortex (G). The higher visual cortex (G) receives feedforward (*W*_*GE*_) input from V1 as well.

To build a neural network with different types of neural connections (feed-forward, recurrent, horizontal, and feedback connections (Figure 1), we firstly constructed a model unit with two local components—local excitatory (E) and inhibitory (I) components [53–56]. The local components E and I can be thought of as a group of neurons recurrently connected within one cortical hypercolumn or a few of such nearby columns in V1.

The dynamic interactions of E and I in local recurrent connection (RC) are described by Equations (1)–(3). The strengths of local interactions between E and I is denoted by *W*_*RS*_, where *R* denotes the receiver and *S* denotes the sender. The interaction type (excitatory or inhibitory) is denoted by the sign of *W*_*RS*_ (positive or negative).

(1) $$\begin{array}{c} \tau_{E}\frac{dE}{dt}=-E+W_{EE}^{RC}H\left(E\right)+W_{EI}^{RC}H\left(I\right)+W_{EL}R_{LGN}, \end{array}$$

(2) $$\begin{array}{c} \tau_{I}\frac{dI}{dt}=-I+W_{IE}^{RC}H\left(E\right)+W_{II}^{RC}H\left(I\right)+W_{IL}R_{LGN}, \end{array}$$

where

(3) $$\begin{array}{c} H\left(x\right)=\left\{\begin{array}{cc} x, & \text{if }x>0\\ 0, & \text{otherwise} \end{array}.\right. \end{array}$$

In the E-I unit, the local E component connects to the local I component with the coupling strength *W*_*IE*_^*RC*^ and connection strength within E components is denoted by *W*_*EE*_^*RC*^. Similarly, the local I component connects to E component with strength *W*_*EI*_^*RC*^ and inhibitory connection within I components is denoted by *W*_*II*_^*RC*^. The *τ*_*E*_ and  *τ*_*I*_ in Equations (1) and (2) are the time constants for E and I, respectively. The local field potential (LFP) is defined as the value of E component in the central position (or in each local unit). “Spiking” thresholds for E and I components are both set to 0 (function H(x) in Equation (3)): only values of E and I that exceed the threshold (i.e., that are greater than zero) will affect other neurons (i.e., H(E)). This can also be viewed as the mean firing rate for a group of E or I neurons. Both E and I receive independent inputs from LGN, the connection strengths between LGN and E or I are denoted as *W*_*EL*_ and *W*_*IL*_. We assume that response from subcortical regions (LGN) as a mean of Gaussian white noise [40], from which we drew a random variable on each time step.

### 2.2. Models with Full Connections

In the network with all connection types, we increased the number of the basic units in the visual cortex horizontally. We added global excitatory (G) component [4, 40, 46] and long-distance horizontal connections (HC). The G component can be thought as higher cortex like V2 or V4 providing feedback connections to V1 E-I units, and the feedback connections are denoted as *W*_*EG*_ and *W*_*IG*_. In this paper, we used 15 × 15 E-I units for a large piece of V1. Beyond the local connection, the local E and I components received the long-distance horizontal connection (HC) *W*_*EE*_^*HC*^ and *W*_*IE*_^*HC*^ from the E component outside of its unit (Equations (4)–(7)). We assumed the local inhibitory component could not directly project with long-range connections to E-I unit outside. The parameters for HC are decayed as Gaussian function with distance (Equation (7)). In the case to investigate the effect of horizontal connections without feedback, we set *W*_*EG*_ = 0, *W*_*IG*_ = 0. In the case to investigate the effect of feedback without horizontal connections, we set *W*_*RE*_^*HC*^ = 0 (R = E or I).

(4) $$\begin{array}{c} \tau_{E}\frac{dE_{i}}{dt}=-E_{i}+W_{EE}^{RC}H\left(E_{i}\right)+W_{EI}^{RC}H\left(I_{i}\right)+W_{EG}H\left(G\right)+\sum W_{EE}^{HC}\left(d\right)H\left(E^{HC}\right)+W_{EL}R_{LGN}, \end{array}$$

(5) $$\begin{array}{c} \tau_{I}\frac{dI_{i}}{dt}=-I_{i}+W_{IE}^{RC}H\left(I_{i}\right)+W_{II}^{RC}H\left(I_{i}\right)+W_{IG}H\left(G\right)+\sum W_{IE}^{HC}\left(d\right)H\left(E^{HC}\right)+W_{IL}R_{LGN}, \end{array}$$

(6) $$\begin{array}{c} \tau_{G}\frac{dG}{dt}=-G+\sum W_{GE}H\left(E_{i}\right), \end{array}$$

(7) $$\begin{array}{c} W_{RE}^{HC}\left(dist\right)=W_{RE}^{HC}\left(\exp\left(-{dist}^{2}/2\sigma^{2}\right)/\sigma \right). \end{array}$$

Where dist denotes for distance between R (local E or I) and global E outside of local E-I unit and *i* equals to 1,2,...225. We set *σ* equals 4. (Figure 1).

### 2.3. Visual Stimuli and Model Inputs

The visual stimuli we used in the simulation throughout the work are considered as the grating. A visual stimulus drives 225 neurons (15 by 15 in visual space same as geometric arrangement for cortical E-I units) in the lateral geniculate nucleus (LGN) and then each LGN neuron will activate its corresponding E-I unit in a network (Figure 1) as described in Equations (1), (2), (4), and (5). For visual stimuli at different size, if a LGN neuron' receptive field is covered by the visual stimulus, its mean firing rate is 40 Hz, and if a LGN neuron's receptive field is outside the visual stimulus (covered by blank), its mean firing rate is set at 0 Hz. The dynamic responses of LGN neurons driven by visual stimuli were modeled as Gaussian white noise (standard deviation is 1) at each time point [40] with its mean firing rate (0 or 40). Corresponding to the stimuli with different sizes, the cortical E-I units within the activation zone with multiple radiuses would receive strong inputs from LGN, and E-I units outsides would receive weak inputs from LGN. The number of E-I units is defined as units within the activation zone, which is approximately proportional to the area of visual stimulus.

### 2.4. Model Simulation

All simulation and data analyses were implemented with custom software written in custom scripts with MATLAB. We solved the equations numerically, with a time resolution of 0.001 second using the Euler method [4, 56]. We ran the model for 1.3 s with 100 repeats for each condition. We analyzed responses starting from 0.3 s after response onset, to ensure the network had settled to a steady state. We used the average half rectified value of E and G (i.e., H(E)) as the mean firing rate (multiunit activity (MUA)), and the power and peak frequency in the power spectrum of E in the central position of the network are defined as the power and peak frequency of LFP, which is a traditional way to model the LFP [4, 56]. The central position means the central E-I unit in the network. The reason for selecting neural activity of central E component as LFP is because the LFP is a local signal [57, 58] and cortical LFP is thought to mainly reflect synaptic activity of excitatory neurons [59, 60].

The value of coupling strengths in all E-I units is identical. We further assumed that the local E has the fastest time constant (6 ms), and the time constant for local I and Global G is slower (12 and 19 ms, respectively). These parameters were fixed for all simulations of stimulus manipulations. The values of coupling strength and *τ*s are provided in Table 1.

### 2.5. Estimation of Gamma Oscillations

We detect the gamma peaks in the LFP power spectrum as follows: firstly, we set a frequency band (taking fast gamma as an example, 45-70 Hz) and find the peak frequency of the strongest power. If its peak frequency is at either of two ends (45 Hz or 70 Hz), we think there is no peak in this band. In this work, we set two frequency bands to search for the peak of slow (25-40 Hz) and fast gamma (45-70 Hz). The method gamma provides a quick way to detect gamma oscillations, and it was also confirmed by eye inspections on all simulation conditions. The value of gamma power is estimated by the subtraction of the maximum power in the frequency band and average power at both ends of the frequency band. In the end, we compared the strength of gamma power and its peak frequency in different stimulus size. The suppression index (SI) of gamma power is defined as the ratio of power under large size to that under optimal size (stimulus size that elicits maximum power). The frequency change index (FC) is defined as the frequency change between the peak frequency induced by the smallest stimulus size and that induced by the largest stimulus size.

## 3. Results

To understand the relationship between gamma oscillation and different types of connections (local recurrent connection: RC; long-distance horizontal connection: HC; feedback connection: FB), we constructed a basic large-scale network with different combinations of local RC, HC, and FB. The basic components in such a network are 225 (15 × 15) E-I units (Figure 1(a)). Each of the E-I unit (Figure 1(b)) consists of a local excitatory component (E) and a local inhibitory component (I), which represents a group of excitatory and inhibitory neurons connected to each other by RC in a local region (one or a few nearby hypercolumns) in V1 (Figure 1(a)). All 225 E-I units are placed horizontally mimicking a piece of macaque V1 with many hypercolumns (Figure 1(a)). Each E-I unit receives external feed-forward (FF) inputs from LGN. When the different local E-I units are connected through long-range horizontal connections (HC), the weights for HC are decayed with distance between two E-I units (Equation (7)). For a network with FB, the G component represents a higher visual cortex, V2/V4, and receives FF input from all E components of the 225 E-I units. The G component forms the feedback connection (FB) to the local E-I units in V1 (Figure 1(b)). The model architectures for FF, RC, HC, and FB are based on existing models for studying gamma oscillation or other functional properties in V1 [17, 40, 61, 62].

### 3.1. HC and Local RC Generate Distinct Gamma Oscillations

**Figure 2:**
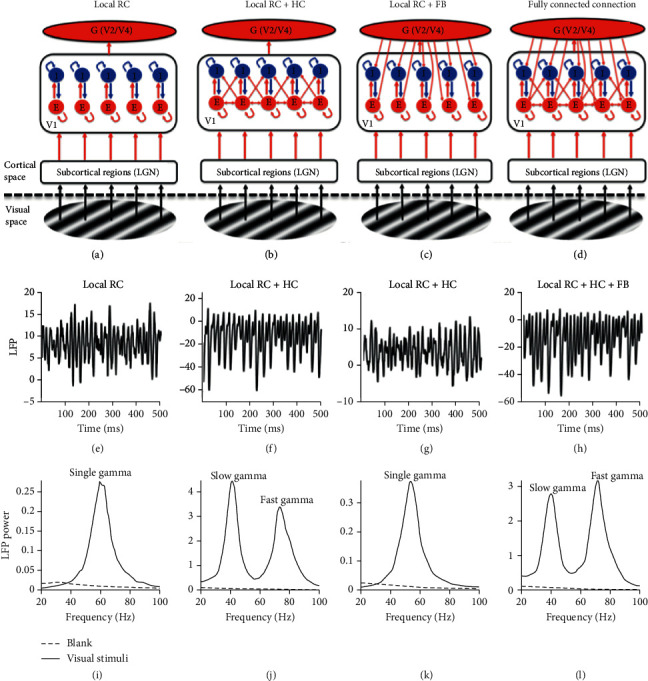
Gamma oscillations generated in networks with different connectivity patterns. Panels (a–d) show networks with different connection patterns. The difference among (a–d) is that (a) shows a network with feedforward (FF) and local connections (local RC); (b) shows a network with local RC and long-range horizontal connection (HC) but without any feedback connection (FB); (c) shows a network with local RC and FB but without HC; and (d) shows a network with local RC, HC, and FB. The local field potentials (LFP) from networks with the four different connection patterns are shown in panels (e–h) correspondingly. The power spectrums of the LFPs were estimated and shown in (i–l) ((i) for the LFP in (e); (j) for that in (f); and (k) for that in (g); and (l) for that in (h)). The black curve shows the power spectrum during the visual stimuli, while the black dashed line shows the power spectrum when networks were driven by a visual stimulus with zero contrast. There is only one gamma in (i) and (k), but two gamma oscillations (slow and fast gamma as noted) appeared in (j) and (l).

The first question we asked is how the three types of connections, RC, HC, and FB, modulate gamma oscillation. To address this question, we formed four networks which contain local RC only (Figure 2(a)), local RC + HC (Figure 2(b)), local RC + FB (Figure 2(c)), and local RC + HC + FB (Figure 2(d)). We drove each of the networks with a large visual stimulus so that all E-I units and their connections can be fully activated. The oscillatory activity could be clearly seen in the LFP from all four networks (local RC for Figure 2(e), local RC + HC for Figure 2(f), local RC + FB for Figure 2(g), and local RC + HC + FB for Figure 2(h)). The corresponding power spectrums further confirm that the four networks can generate gamma oscillations (Figures 2(i)–2(m)). Consistent with early work [4, 40, 56], a single gamma oscillating at 59 Hz could be generated in network with only local RC circuits (Figure 2(i)) or with local RC and FB (Figure 2(k)). Surprisingly, when HC was added into the network, even though RC and FF strength were unchanged, a slow gamma oscillating at 41 Hz emerges (Figures 2(j) and 2(l)). It is notable that the slow gamma oscillation is a new gamma oscillation, because its peak frequency is not the harmonic of the fast gamma (73 Hz) generated by RC (Figure 2(j)). Interestingly, when we added FB to the network and removed HC (Figure 2(c)), the slow gamma disappeared and only a single gamma exists (Figure 2(k)) but with its peak frequency (53 Hz) lower than that generated in the network with only FF and RC (Figure 2(i)). When we added both FB and HC (Figure 2(d)), the slow gamma emerges again oscillating at 40 Hz and fast gamma oscillates in 71 Hz (Figure 2(l)).

Model simulations in this section suggest that HC can generate a slow gamma that is different from the fast gamma generated by local RC. However, FB itself cannot generate a new gamma. This conclusion from our simulation results can be further proved mathematically.

### 3.2. Mathematic Evidence for Two Gamma Oscillations in a Network with HC and RC

To understand the generation of two distinct gamma oscillations in the network with HC mathematically, let us first consider the network with FB only, which can be described and abbreviated as Equations (8)–(10).

(8) $$\begin{array}{c} \tau_{E}\frac{dE_{i}}{dt}=-E_{i}+W_{EE}^{RC}H\left(E_{i}\right)+W_{EI}^{RC}H\left(I_{i}\right)+W_{EG}H\left(G\right)+W_{EL}R_{LGN}, \end{array}$$

(9) $$\begin{array}{c} \tau_{I}\frac{dI_{i}}{dt}=-I_{i}+W_{IE}^{RC}H\left(E_{i}\right)+W_{II}^{RC}H\left(I_{i}\right)+W_{IG}H\left(G\right)+W_{IL}R_{LGN}, \end{array}$$

(10) $$\begin{array}{c} \tau_{G}\frac{dG}{dt}=-G+\sum W_{GE}H\left(E_{i}\right), \end{array}$$

where *i* equals to 1,2,...225. Equations (8) and (9) are an abbreviation for 450 equations for 225 E-I units, and Equation (10) is for the G component.

Now, let us assume that *E* = ∑*E*_*i*_, *I* = ∑*I*_*i*_ and *H*(*x*) = *x*. Then, we can get Equations (11)–(13).

(11) $$\begin{array}{c} \tau_{E}\frac{dE}{dt}=\tau_{E}\frac{d\sum E_{i}}{dt}=\frac{\sum \tau_{E}dE_{i}}{dt}=\sum \left(-E_{i}+W_{EE}^{RC}H\left(E_{i}\right)+W_{EI}^{RC}H\left(I_{i}\right)+W_{EG}H\left(G\right)+W_{EL}R_{LGN}\right)=-E+W_{EE}^{RC}E+W_{EI}^{RC}I+nW_{EG}G+\sum W_{EL}R_{LGN}, \end{array}$$

(12) $$\begin{array}{c} \tau_{I}\frac{dI}{dt}=\tau_{I}\frac{d\sum I_{i}}{dt}=\frac{\sum \tau_{I}dI_{i}}{dt}=\sum \left(-I_{i}+W_{IE}^{RC}H\left(E_{i}\right)+W_{II}^{RC}H\left(I_{i}\right)+W_{IG}H\left(G\right)+W_{IL}R_{LGN}\right)=-I+W_{IE}^{RC}E+W_{II}^{RC}I+nW_{IG}G+\sum W_{IL}R_{LGN}, \end{array}$$

(13) $$\begin{array}{c} \tau_{G}\frac{dG}{dt}=-G+\sum W_{GE}H\left(E_{i}\right)=-G+nW_{GE}E. \end{array}$$

In this way, we compressed 451 equations into a three-dimensional system. We can see that any solution for the original 451 equations will also satisfy the three-dimensional system. In other words, solutions for the three-dimensional system should be exactly solutions for the original 451-dimensional system. Notice that this is true for a FB-only-system with any number (n) of E-I units.

The weight matrix *W*_*FB*_ could be written in Equation (14) (*n* = 225):

(14) $$\begin{array}{c} W_{FB}=\left(\begin{array}{ccc} \frac{1}{\tau_{E}}\left(W_{EE}^{RC}-1\right) & \;\frac{1}{\tau_{E}}W_{EI}^{RC}\; & \frac{n}{\tau_{E}}W_{GE}\\ \frac{1}{\tau_{I}}W_{IE}^{RC} & \frac{1}{\tau_{I}}\left(W_{II}^{RC}-1\right) & \frac{n}{\tau_{I}}W_{GE}\\ \frac{n}{\tau_{G}}W_{GE} & 0\; & -\frac{1}{\tau_{G}} \end{array}\right). \end{array}$$

The eigenvalues of the matrix *W*_*FB*_ could be classified into two types: (1) three real eigenvalues: it means this system does not generate any oscillation. (2) One real and two complex eigenvalues: it means this system could generate one oscillation. From the simple mathematical derivation, we found that the feedback connection in the brain system does not have the mathematical basis to generate distinct gamma oscillations.

Similarly, let us consider the network with HC only, which could be written in the abbreviated form of Equations (15) and (16) for 450 original equations.

(15) $$\begin{array}{c} \tau_{E}\frac{dE_{i}}{dt}=-E_{i}+W_{EE}^{RC}H\left(E_{i}\right)+W_{EI}^{RC}H\left(I_{i}\right)+\underset{j\neq i}{\sum }W_{E_{i}E_{j}}^{HC}H\left(E_{j}\right)+W_{EL}R_{LGN}, \end{array}$$

(16) $$\begin{array}{c} \tau_{I}\frac{dI_{i}}{dt}=-I_{i}+W_{IE}^{RC}H\left(E_{i}\right)+W_{II}^{RC}H\left(I_{i}\right)+\underset{j\neq i}{\sum }W_{I_{i}E_{j}}^{HC}H\left(E_{j}\right)+W_{IL}R_{LGN}, \end{array}$$

where *i* equals to 1,2,...225, *W*_*E*_*i*_*E*_*j*__^*HC*^ = *αW*_*I*_*i*_*E*_*j*__^*HC*^, *α* is a constant.

Now, let us assume that *E*_*HC*_ = ∑_*j*≠*i*_*W*_*E*_*i*_*E*_*j*__^*HC*^*E*_*j*_, *E*_*RC*_ = ∑_*i*=1_^*N*^*E*_*i*_, *I*_*HC*_ = ∑_*j*≠*i*_*W*_*I*_*i*_*E*_*j*__^*HC*^*I*_*j*_, *I*_*RC*_ = ∑_*i*=1_^*N*^*I*_*i*_

and *H*(*x*) = *x*. Then, we got Equations (17)–(22)

(17) $$\begin{array}{c} \tau_{E}\frac{dE_{RC}}{dt}=\overset{N}{\underset{i=1}{\sum }}\left(-E_{i}+W_{EE}^{RC}\left(E_{i}\right)+W_{EI}^{RC}\text{H}\left(I_{i}\right)+\underset{j\neq i}{\sum }W_{E_{i}E_{j}}^{HC}H\left(E_{j}\right)+W_{EL}R_{LGN}\right)=-E_{RC}+W_{EE}^{RC}E_{RC}+W_{EI}^{RC}I_{RC}+\overset{N}{\underset{i=1}{\sum }}\underset{j\neq i}{\sum }W_{E_{i}E_{j}}^{HC}H\left(E_{j}\right)+\overset{N}{\underset{i=1}{\sum }}W_{EL}R_{LGN}, \end{array}$$

(18) $$\begin{array}{c} \tau_{E}\frac{dI_{RC}}{dt}=\overset{N}{\underset{i=1}{\sum }}\left(-I_{i}+W_{IE}^{RC}\left(E_{i}\right)+W_{II}^{RC}H\left(I_{i}\right)+\underset{j\neq i}{\sum }W_{I_{i}E_{j}}^{HC}H\left(E_{j}\right)+W_{IL}R_{LGN}\right)=-{\text{I}}_{RC}+W_{IE}^{RC}E_{RC}+W_{II}^{RC}I_{RC}+\overset{N}{\underset{i=1}{\sum }}\underset{j\neq i}{\sum }{\alpha W}_{E_{i}E_{j}}^{HC}H\left(E_{j}\right)+\overset{N}{\underset{i=1}{\sum }}W_{IL}R_{LGN}, \end{array}$$

where ∑_*i*=1_^*N*^∑_*j*≠*i*_*W*_*E*_*i*_*E*_*j*__^*HC*^*H*(*E*_*j*_) ≈ *NE*_*HC*_, *N* = 225.

(19) $$\begin{array}{c} \tau_{E}\frac{dE_{HC}}{dt}=\tau_{E}\frac{\sum_{j\neq i}W_{E_{i}E_{j}}^{HC}{dE}_{j}\;}{dt}=\underset{j\neq i}{\sum }W_{E_{i}E_{j}}^{HC}\;\tau_{E}\frac{dE_{j}}{dt}=\underset{j\neq i}{\sum }W_{E_{i}{\text{E}}_{j}}^{HC}\left(-E_{j}+W_{EE}^{RC}H\left(E_{j}\right)+W_{EI}^{RC}H\left(I_{j}\right)+\underset{k\neq j}{\sum }W_{E_{j}E_{k}}^{HC}H\left(E_{k}\right)+W_{EL}R_{LGN}\right)=-E_{HC}+W_{EE}^{RC}E_{HC}+W_{EI}^{RC}I_{HC}+\underset{j\neq i}{\sum }W_{E_{i}E_{j}}^{HC}\;\underset{k\neq j}{\sum }W_{E_{j}E_{k}}^{HC}E_{k}+W_{FF\left(E\right)}R_{LGN}, \end{array}$$

where *W*_*FF*(*E*)_ = ∑_*j*≠*i*_*W*_*E*_*i*_*E*_*j*__^*HC*^ *W*_*EL*_

(20) $$\begin{array}{c} \underset{j\neq i}{\sum }W_{E_{i}E_{j}}^{HC}\;\underset{k\neq j}{\sum }W_{E_{j}E_{k}}^{HC}E_{k}=\underset{j\neq i}{\sum }W_{E_{i}E_{j}}^{HC}\;\left(W_{E_{j}E_{1}}^{HC}E_{1}+W_{E_{j}E_{2}}^{HC}E_{2}+\cdots +W_{E_{j}E_{N}}^{HC}E_{N}\right)\\ \underset{j\neq i}{\sum }W_{E_{i}E_{j}}^{HC}\;W_{E_{j}E_{k}}^{HC}E_{k}\approx \frac{E_{k}\iint {W_{EE}^{HC}}^{2}e^{-{\left(x_{i}-x_{j}\right)}^{2}+{\left(y_{i}-y_{j}\right)}^{2}+{\left(x_{k}-x_{j}\right)}^{2}+{\left(y_{k}-y_{j}\right)}^{2}/2\sigma^{2}}}{\sigma^{2}}d\left(x_{j}\right)d\left(y_{j}\right)=E_{k}{W_{EE}^{HC}}^{2}\iint e^{-{\left(\frac{x_{j}-\left(x_{k}+x_{i}\right)}{\sigma }\right)}^{2}}e^{-{\left(\frac{y_{j}-\left(y_{k}+y_{i}\right)}{\sigma }\right)}^{2}}P\left(k,i\right)d\left(\frac{x_{j}}{\sigma }\right)d\left(\frac{y_{j}}{\sigma }\right), \end{array}$$

where *P*(*k*, *i*) = *e*^−(*x*_*k*_^2^ + *y*_*k*_^2^ + *x*_*i*_^2^ + *y*_*i*_^2^ − 2*x*_*k*_*x*_*i*_ − 2*y*_*k*_*y*_*i*_)/4*σ*^2^^ = *e*^−[(*x*_*k*_ − *x*_*i*_)^2^ + (*y*_*k*_ − *y*_*i*_)^2^]/4*σ*^2^^, (*x*_*i*_, *y*_*i*_) is the coordination of *E*_*i*_.

Hence, ∑_*j*≠*i*_*W*_*E*_*i*_*E*_*j*__^*HC*^ *W*_*E*_*j*_*E*_*k*__^*HC*^*E*_*k*_ = *E*_*k*_*W*_*EE*_^*HC*^^2^*P*(*k*, *i*) *π*

(21) $$\begin{array}{c} \underset{j\neq i}{\sum }W_{E_{i}E_{j}}^{HC}\;\underset{k\neq j}{\sum }W_{E_{j}E_{k}}^{HC}E_{k}=\overset{N}{\underset{k=1}{\sum }}E_{k}{W_{EE}^{HC}}^{2}P\left(k,i\right)\;\pi =\overset{N}{\underset{k=1}{\sum }}E_{k}{W_{EE}^{HC}}^{2}e^{-\left[{\left(x_{k}-x_{i}\right)}^{2}+{\left(y_{k}-y_{i}\right)}^{2}\right]/4\sigma^{2}}\;\pi =\frac{{W_{EE}^{HC}}^{2}\pi \sum_{k=1}^{N}e^{-\left[{\left(x_{k}-x_{i}\right)}^{2}+{\left(y_{k}-y_{i}\right)}^{2}\right]/4\sigma^{2}}\;E_{k}\approx {W_{EE}^{HC}}^{2}\pi \sum_{k=1}^{N}e^{-\left[{\left(x_{k}-x_{i}\right)}^{2}+{\left(y_{k}-y_{i}\right)}^{2}\right]/4\sigma^{2}}\;\sum_{n=1}^{N}\;E_{n}}{N}\approx \frac{{W_{EE}^{HC}}^{2}\pi }{N}E_{LC}\iint e^{-\frac{\left[{\left(x_{k}-x_{i}\right)}^{2}+{\left(y_{k}-y_{i}\right)}^{2}\right]}{4\sigma^{2}}}dx_{k}dy_{k}=\frac{4\sigma^{2}{W_{EE}^{HC}}^{2}\pi^{2}}{N}E_{RC}. \end{array}$$

Similarly,

(22) $$\begin{array}{c} \tau_{I}\frac{dI_{HC}}{dt}=\tau_{I}\frac{\sum_{j\neq i}W_{E_{i}E_{j}}^{HC}{dI}_{j}\;}{dt}=\underset{j\neq i}{\sum }W_{E_{i}E_{j}}^{HC}\;\tau_{I}\frac{dI_{j}}{dt}=\underset{j\neq i}{\sum }W_{E_{i}E_{j}}^{HC}\;\left(-I_{j}+W_{IE}^{RC}H\left(E_{j}\right)+W_{II}^{RC}H\left(I_{j}\right)+\underset{k\neq j}{\sum }W_{I_{i}E_{j}}^{HC}H\left(E_{k}\right)+W_{IL}R_{LGN}\right)=-I_{HC}+W_{EE}^{RC}E_{HC}+W_{EI}^{RC}I_{HC}+\alpha \underset{j\neq i}{\sum }W_{E_{i}E_{j}}^{HC}\;\underset{k\neq j}{\sum }W_{E_{j}E_{k}}^{HC}H\left(E_{k}\right)+W_{FF\left(I\right)}R_{LGN}, \end{array}$$

where *W*_*FF*(*I*)_ = ∑_*j*≠*i*_*W*_*E*_*i*_*E*_*j*__^*HC*^ *W*_*IL*_.

Notice that connection weights of HC (*W*_*R*_*i*_*E*_*j*__^*HC*^) are related to the distance between different E-I units (Equation (7)). If the sigma parameter (*σ*) in Equation (7) is large enough, Equations (19) and (22) could be rewritten as Equations (23) and (24).

(23) $$\begin{array}{c} \tau_{E}\frac{dE_{HC}}{dt}=-E_{HC}+4\sigma^{2}{W_{EE}^{\text{H}C}}^{2}\pi^{2}E_{RC}+W_{EE}^{RC}E_{HC}+W_{EI}^{RC}I_{HC}+W_{FF\left(E\right)}R_{LGN}, \end{array}$$

(24) $$\begin{array}{c} \tau_{I}\frac{dI_{HC}}{dt}=-I_{HC}+4\alpha \sigma^{2}{W_{EE}^{HC}}^{2}\pi^{2}E_{RC}+W_{IE}^{RC}E_{HC}+W_{II}^{RC}I_{HC}+W_{FF\left(I\right)}R_{LGN}. \end{array}$$

In this way, we compressed 450 equations for HC network into a four-dimensional system. Again, we can see that solutions for the four-dimensional system should be exactly solutions for the original 450-dimensional system. Notice that this is true for an HC-only-system with any number (n) of E-I units, when spatial constant (*σ*) for HC connection is large enough.

Then, the weight matrix *W*_*HC*_ could be written in Equation (25):

(25) $$\begin{array}{c} W_{HC}=\left(\begin{array}{cccc} \frac{1}{\tau_{E}}\left(W_{EE}^{RC}-1\right) & \frac{1}{\tau_{E}}W_{EI}^{RC} & \frac{N}{\tau_{E}} & 0\\ \frac{1}{\tau_{I}}W_{IE}^{RC} & \frac{1}{\tau_{I}}\left(W_{II}^{RC}-1\right) & \frac{\alpha N}{\tau_{I}} & 0\\ \frac{4\sigma^{2}{W_{EE}^{HC}}^{2}\pi^{2}}{N} & 0 & \;\frac{1}{\tau_{E}}\left(W_{EE}^{RC}-1\right) & \frac{1}{\tau_{E}}W_{EI}^{RC}\\ \;\alpha \frac{4\sigma^{2}{W_{EE}^{HC}}^{2}\pi^{2}}{N} & 0 & \frac{1}{\tau_{I}}W_{EE}^{RC} & \frac{1}{\tau_{I}}\left(W_{II}^{RC}-1\right) \end{array}\right). \end{array}$$

The eigenvalues of the matrix *W*_*HC*_ could be classified into three types: (1) four real eigenvalues: it means this system does not generate any oscillation; (2) two real and two complex eigenvalues: it means this system could generate one oscillation; (3) four complex eigenvalues: it means this system could generate two different oscillations. The conjugate complex eigenvalues could be written in the form of *λ* = *a* ± *bi*, where *a* and *b* are the real and imaginary part of the complex value. The peak frequency of the oscillation generated by this system could be equaled to *b*/2*π*. When the sigma parameter in Equation (7) is small, the system will be more complex, and in theory, oscillations in the system could be more than two. But as our simulation shows, within our parameter space, we only observed one or two gamma oscillations, which suggests that the spatial constant (*σ*) is large.

The above mathematical derivation provides a conceptual picture and fundamental evidence for why a network with local RC + HC can generate two gamma oscillations but local RC + FB cannot. However, the derivation, based on simplified and linearized equations of the original nonlinear dynamic systems, does not give us detailed parameter regimes (*W*_*EE*_^*HC*^ or *W*_*IE*_^*HC*^, the long-range horizontal connection from E to E and I, respectively) for the generation of the slow gamma. We went back to model simulation to further understand the functional roles of *W*_*EE*_^*HC*^ and *W*_*IE*_^*HC*^ in the network with HC.

### 3.3. Inhibitory Strength in HC Determines the Number of Gamma Oscillations

**Figure 3:**
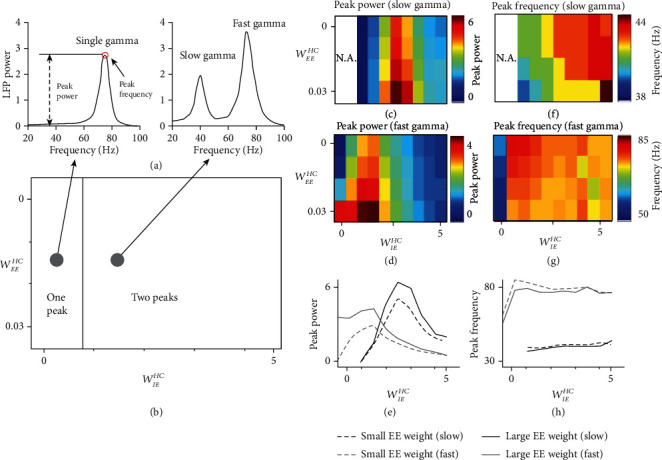
Phase diagram for the number of gamma oscillations and the modulation of existing gamma in HC network without FB. (a) shows the power spectrums of the LFPs from two example networks. Although both of the two networks have local RC and HC, but their HC connection strengths are different. There are two distinct oscillatory states (b): (1) one gamma oscillation and (2) two gamma oscillations. These oscillatory states are dependent on parameters (*W*_*EE*_^*HC*^ and *W*_*IE*_^*HC*^) for HC connection strengths of the network in (b). The power of slow and fast gamma for different connection strength is shown in (c) and (d), respectively. (e) shows how the peak power changes with connection weight *W*_*IE*_^*HC*^ corresponding to color plots (c) and (d). The black lines denote the cases of slow gamma (dashed black line for *W*_*EE*_^*HC*^ = 0, and solid black line for *W*_*EE*_^*HC*^ = 0.03), and grey lines are for fast gamma (dashed grey line is for *W*_*EE*_^*HC*^ = 0, and solid grey line is for *W*_*EE*_^*HC*^ = 0.03). The peak frequency of slow and fast gamma for different connection strength is shown in (f) and (g), respectively. (h) shows how peak frequency changes of connection weight *W*_*IE*_^*HC*^ corresponding to the color plot in (c) and (d). The black line denotes the case of slow gamma (dashed line for *W*_*EE*_^*HC*^ = 0, and solid line for *W*_*EE*_^*HC*^ = 0.03) and grey line is for that of fast gamma (dashed line for *W*_*EE*_^*HC*^ = 0, and solid line for *W*_*EE*_^*HC*^ = 0.03).

We went through parameter values for *W*_*EE*_^*HC*^ and *W*_*IE*_^*HC*^ in a reasonable range (*W*_*EE*_^*HC*^: 0-0.03, *W*_*IE*_^*HC*^: 0-4.5) and simulated network models with different combinations of *W*_*EE*_^*RC*^ and *W*_*IE*_^*RC*^. We found two states in the parameter ranges for model simulation: (1) when *W*_*IE*_^*HC*^ value is small (0-0.75), there is only a single gamma, and HC circuitry does not generate the second gamma (Figures 3(a) (left) and 3(b)); (2) when *W*_*IE*_^*HC*^ value is in a range of medium values (0.75-4.5), both fast gamma and slow gamma coexist (Figures 3(a) (right) and 3(b)). The number of gamma oscillations in a network is clearly dependent on the connection strength of *W*_*IE*_^*HC*^, but not *W*_*EE*_^*HC*^ (Figure 3(b)). In the current study, we mainly focused on the two states for network oscillations.

Next, we aimed to explore how these two gamma oscillations are modulated through HC. We defined two indexes: peak power (the max power in the specific gamma frequency band) and peak frequency (the frequency that achieves peak power in the specific gamma frequency band) as shown in Figure 3(a). The peak power for slow gamma increases with *W*_*IE*_^*HC*^ (from 1 to 2.5) and then decreases with *W*_*IE*_^*HC*^ (from 2.5 to 4) (Figure 3(c)), and the power of fast gamma has a similar relationship with *W*_*IE*_^*HC*^ but in different parameter range (increasing with *W*_*IE*_^*HC*^ from 0 to 1.5 and decreasing with *W*_*IE*_^*HC*^ from 1.5 to 4.5) (Figure 3(d) and (e)). While as the increase of *W*_*EE*_^*HC*^, peak powers of both slow and fast gamma are increasing (Figure 3(c) and (d)). Interestingly, the peak frequency for slow gamma increases with *W*_*IE*_^*HC*^ (Figure 3(f)), but frequency of fast gamma decreases with *W*_*IE*_^*HC*^ (from 0.5 to 4.5), except that there is a rapid rise of frequency for small *W*_*IE*_^*HC*^ from 0 to 0.5, (Figure 3(g)). This shows that HC modulates the peak frequency of slow and fast gamma oscillations in different ways (Figure 3(h)). It is also noted that the range of *W*_*EE*_^*HC*^ is much smaller compared to that of *W*_*IE*_^*HC*^. This is because a large value of *W*_*EE*_^*HC*^ will lead to instability of the network, and we could already see a quick increase of gamma power in the very narrow range of *W*_*EE*_^*HC*^ (Figures 3(c) and (d)).

### 3.4. Modulation of Gamma Oscillations by FB

The simulation results (Figure 3) suggest that the generation of slow gamma oscillation mainly depends on the HC connection from excitatory neurons to inhibitory neurons (*W*_*IE*_^*HC*^), and HC connection from excitatory neurons to excitatory neurons (*W*_*EE*_^*HC*^) mainly modulates existing fast and slow gamma oscillations. In the real brain, feedback connection (FB) is also indispensable. To further understand how existing gamma oscillations are modulated through FB, we fixed the model parameters of HC (*W*_*IE*_^*HC*^ = 2.5, *W*_*EE*_^*HC*^ = 0.03) that can generate two gamma oscillations and added connections (*W*_*EG*_, *W*_*IG*_) from the global excitatory component (G) to local excitatory (E) and inhibitory (I) component.

**Figure 4:**
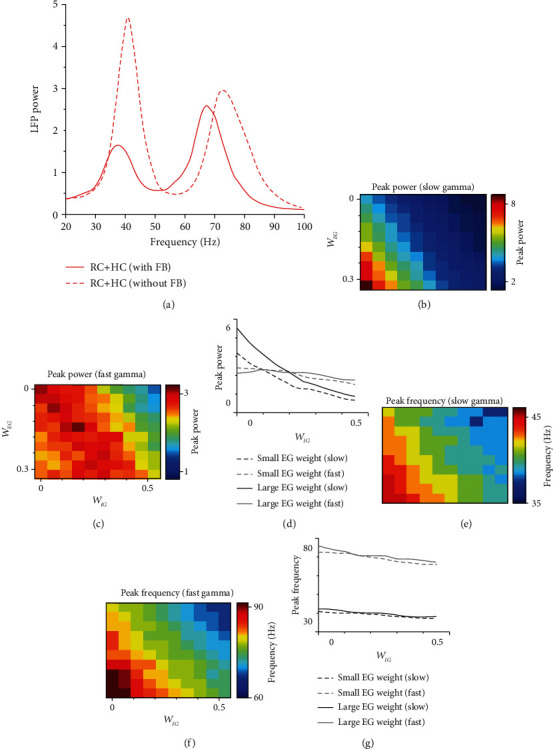
Modulation of gamma oscillations in HC network with FB. (a) shows the power spectrum of the LFPs from two example networks (solid red curve is the power spectrum from the network with FB, and the dashed red curve is from the network without FB). (b–g) are similar to Figures 3(c)–3(h). The powers of slow and fast gamma for different FB connection strength are shown in (b) and (c), respectively. (d) shows how peak power changes with connection weight *W*_*IG*_ at two *W*_*EG*_values (similar to color plots (b) and (c)). The black line denotes the case of slow gamma (dashed black line for *W*_*EG*_ = 0, and solid black line for *W*_*EG*_ = 0.09), and grey line is for that of fast gamma (dashed grey line for *W*_*EG*_ = 0, and solid grey line for *W*_*EG*_ = 0.09). The peak frequency of slow and fast gamma for different connection strength is shown in (e) and (f), respectively. (g) shows how peak frequency changes of connection weight *W*_*IG*_ corresponding to the color plot in (c) and (d). The black line denotes the case of slow gamma (dashed black line for *W*_*EG*_ = 0, and solid black line for *W*_*EG*_ = 0.09), and grey line is for that of fast gamma (dashed grey line for *W*_*EG*_ = 0, and solid grey line for *W*_*EG*_ = 0.09).

**Figure 5:**
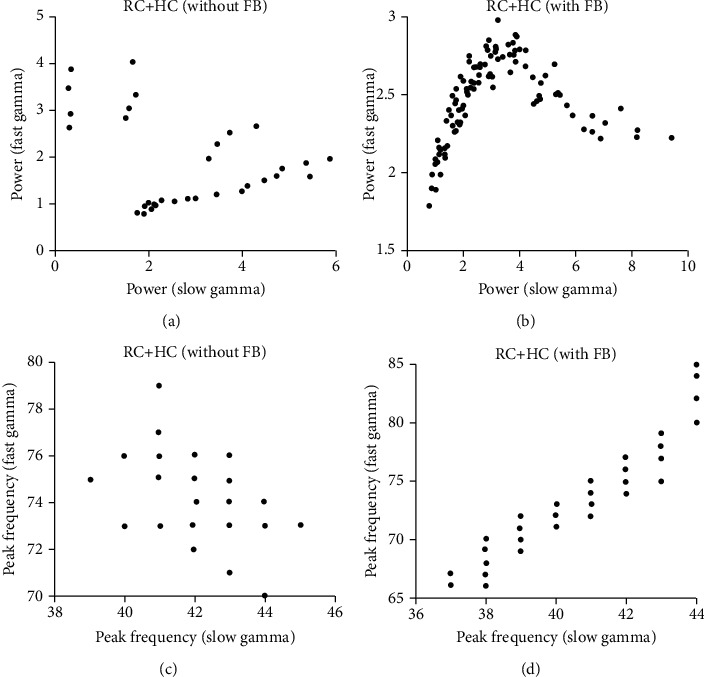
Relationship between gamma oscillations in the HC network without and with FB. (a) shows the scatter plot of peak power for slow (*x*-axis) and fast gamma (*y*-axis) in the network without FB. (b) shows the scatter plot of peak power for slow (*x*-axis) and fast gamma (*y*-axis) in the network with FB. (c) shows the scatter plot of peak frequency for slow (*x*-axis) and fast gamma (*y*-axis) in the network without FB. (d) shows the scatter plot of peak frequency for slow (*x*-axis) and fast gamma (*y*-axis) in the network with FB. The data points for (a) and (c) are peak powers and frequencies in different weights of HC, and for (b) and (d) are that in different weights of FB.

As expected, the FB could modulate the response property of gamma oscillations (Figure 4(a)). Interestingly, both slow and fast gamma oscillations are modulated by FB in a similar way, which is totally different from HC modulation on gamma. Similar to Figures 3(c)–(h), we went through parameter values for *W*_*EG*_ and *W*_*IG*_ in a reasonable range (*W*_*EG*_: 0-0.27; *W*_*IG*_: 0-0.45), and simulated network model with different combinations of *W*_*EG*_ and *W*_*IG*_. The peak power and frequency for both slow and fast gamma increase as the *W*_*EG*_ increases, but the two features decrease as *W*_*IG*_ increases (Figures 4(b)–4(g)). We further compared effect difference in network with and without FB (Figure 5). We found that change of peak power and frequency without FB is both unbalanced and discontinuous (Figures 5(a) and 5(c)); however, FB had a more balanced and consistent effect on the change of peak power and frequency in the HC network with FB (Figures 5(b) and 5(d)).

### 3.5. Stimulus Size Dependence of Gamma Oscillations in the Network with Both HC and FB

**Figure 6:**
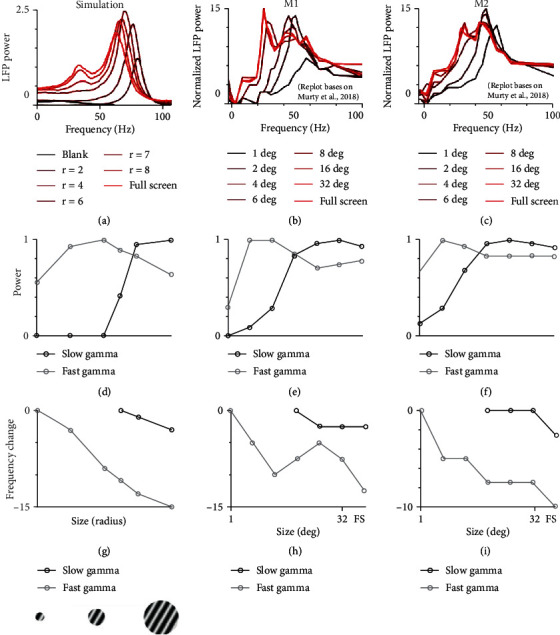
The size dependence of gamma in the model matches experimental results. (a) shows power spectrums of the LFP induced by stimuli at different sizes in our fully connected network. Different stimulus sizes are labelled in different colors. (b) and (c) show how power spectrums (normalized by the response of blank stimuli) change with stimulus sizes in V1 of two monkeys M1 and M2 (Murty et al. 2018). Note: (b) and (c) are replotted based on a recent publication (Murty et al. 2018). (d–f) shows the tuning curves of stimulus size from model simulation, monkey M1 and M2, respectively. The peak value in each tuning curve was normalized peak power for slow (black curve) and fast gamma (grey curve). (g–i) show the tuning curves of stimulus size from model simulation, monkey M1 and M2, respectively. The peak frequency was shown as frequency relative to the highest frequency for slow (black curve) and fast gamma (grey curve).

We have shown that how response properties of gamma oscillations were affected by the change of connection weights of HC and FB. In reality, however, the connections between neurons are almost fixed in the brain. A more realistic way to perturb the connection weights of neural network is to change the network's visual stimuli. The experiment with different size of visual stimuli is commonly used to activate neurons and their connections in different spatial scales in visual cortex [30, 38, 63, 64]. Recently, an experimental work showed that large visual stimuli could induce two distinct gamma oscillations [65] which is highly consistent with our model's prediction (Figure 6). We next explored how gamma oscillations are modulated by stimulus size. A typical example shows that the two gamma oscillations behave differently when stimulus size changes from the simulation (Figure 6(a)). The fast gamma oscillation appears in a much smaller size, while the slow gamma emerges when stimulus size is around 6 (radius) and above (Figure 6(a)). This is highly consistent with the experimental result (Figures 6(b) and 6(c)). The tuning curves for peak power and frequency change (frequency change between the peak frequency induced by the smallest stimulus size and that induced by the largest stimulus size) are also very similar in quantity (Figures 6(d)–6(i)).

**Figure 7:**
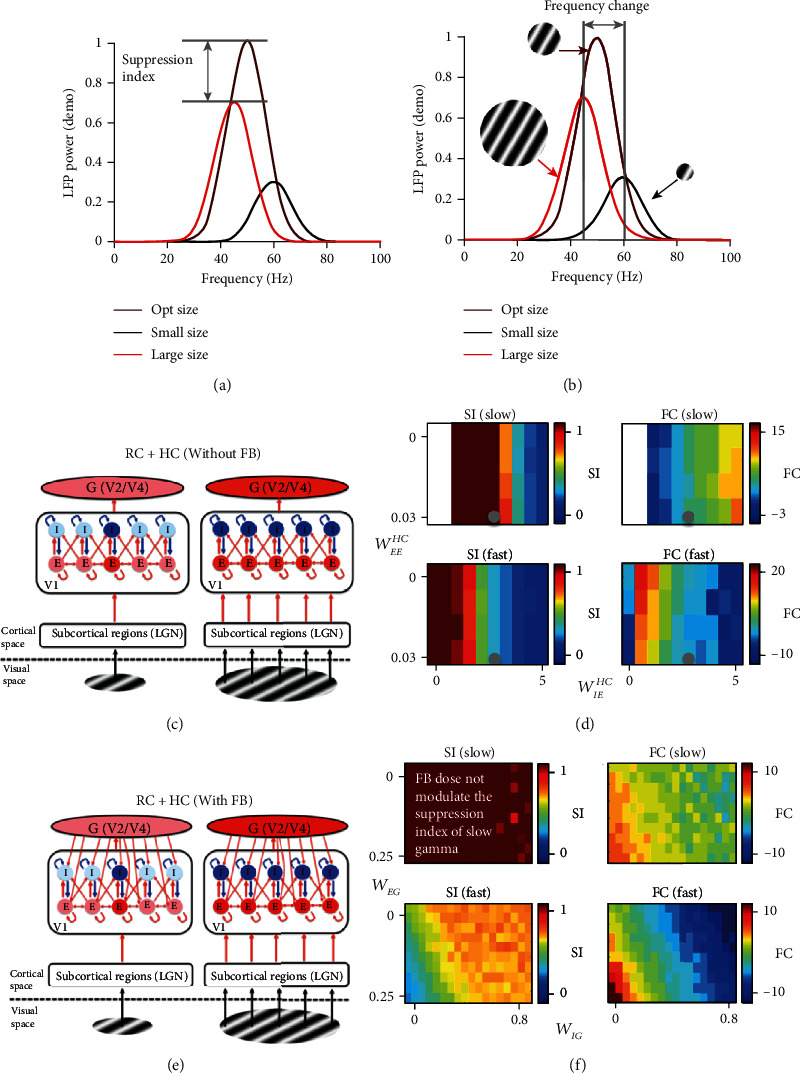
Size dependence of gamma in the HC network with and without FB. The calculation of two indexes (suppression index (SI) and frequency change (FC)) that capture the size-dependent property of gamma was shown in (a) (suppression index) and (b) (frequency change). The curves in different colors mean the power spectrums under different stimulus sizes. (c) shows an HC network without FB driven by a small stimulus (left panel) and by a large stimulus (right panel). Components weakly or not activated by the stimulus are shown as light red or light blue color and components in red or blue are those strongly activated by the stimulus. The left column of (d) shows how the suppression index changes with strengths of *W*_*EE*_^*HC*^ and *W*_*IE*_^*HC*^ for slow (upper panel) and fast gamma (lower panel) oscillation, and the right column of (d) shows how gamma frequency changes with strengths of *W*_*EE*_^*HC*^ and *W*_*IE*_^*HC*^ for slow (upper) and fast gamma (lower) oscillation. (e) shows a network with both HC and FB driven by a small stimulus (left panel) and by a large stimulus (right panel). Notice that the network in (e) and (c) are the same, except that network in (e) has FB, but the network in (c) does not. The panels in (f) are also similar to those in (d), but the *x*-axis and *y*-axis is for strengths of *W*_*EG*_ and *W*_*IG*_ instead.

We have shown that our fully connected model could replicate the size dependence of gamma oscillations in the real experiment. The final question for us in this study is whether FB is a required component for explaining experimental results for size tuning curve [65]. We measured the suppression index of gamma power by calculating the ratio of power under large size to that under optimal size (stimulus size that elicits maximum power) (Figure 7(a)), and frequency change index by subtraction of the peak frequency from the size could induce the gamma and largest size (Figure 7(b)).

Then, we went through different values in a range for parameters *W*_*EE*_^*HC*^ and *W*_*IE*_^*HC*^ for the network of RC + HC without FB (Figure 7(c)) and created maps (Figure 7(d)) for suppression index and frequency change for slow and fast gamma as functions of HC parameters *W*_*EE*_^*HC*^ and *W*_*IE*_^*HC*^. Next, we added the feedback connection in the model (Figure 7(e)) and kept the HC parameters the same as previous settings (Figure 7(d) grey circle) that could generate two gamma oscillations. Then, we also created maps (Figure 7(f)) for suppression index and frequency change for slow and fast gamma as functions of FB parameters *W*_*EG*_ and *W*_*IG*_. It is very clear that FB has different modulatory effects on the size-dependent behaviors of slow and fast gamma (Figures 7(d) and 7(f)).

**Figure 8:**
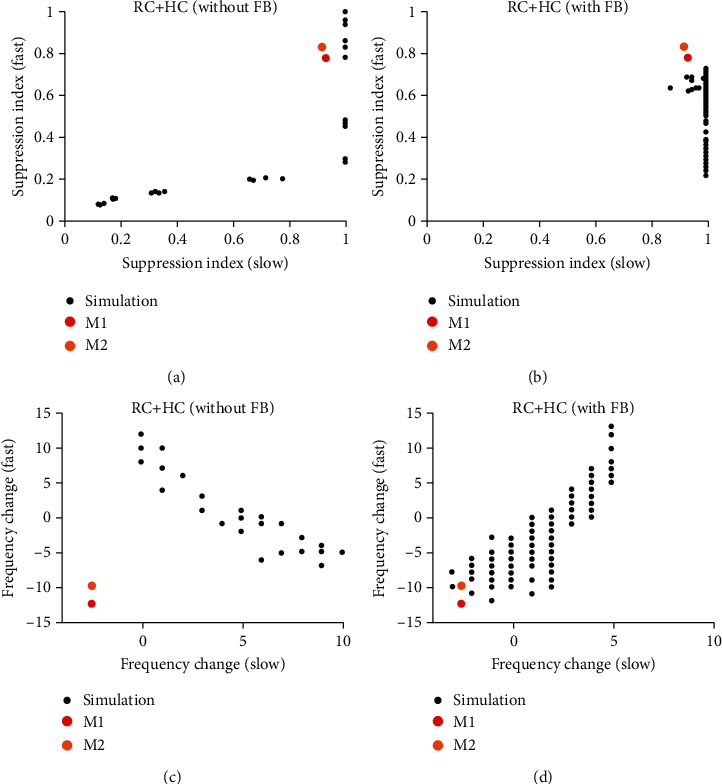
Gamma oscillations in V1 require both HC and FB. (a) shows the scatter plot of suppression index for slow (*x*-axis) and fast gamma (*y*-axis) in the network without FB. (b) shows the scatter plot of suppression index for slow (*x*-axis) and fast gamma (*y*-axis) in the network with FB. (c) shows the scatter plot of frequency change for slow (*x*-axis) and fast gamma (*y*-axis) in the network without FB. (d) shows the scatter plot of frequency change for slow (*x*-axis) and fast gamma (*y*-axis) in the network with FB.

To further illustrate how FB and HC affect gamma oscillations differently as functions of stimulus sizes, scatter plots were plotted for suppression index of slow and fast gamma in the network without FB (Figure 8(a)) and in the network with FB (Figure 8(b)). Similar scatter plots for frequency change of slow and fast gamma were also shown in Figures 8(c) and 8(d), respectively.

In the network without FB, suppression index for slow and fast gamma is positively correlated (Figure 7(d), left column; also see Figure 8(a)), but frequency changes of slow and fast are negatively correlated (Figure 7(d), right column; and Figure 8(d)). Different from the network without FB, we found that FB has minor effect on suppression index of slow gamma (Figure 7(f), left column; Figure 8(b)). Very interestingly, the patterns of frequency change for slow and fast gamma are positively correlated and highly consistent (Figure 7(f), right column; Figure 8(d)), which is very different from HC effects (Figure 7(d) and Figure 8(c)).

More importantly, we found that FB is a necessary component for regulating size-dependent behaviors of gamma oscillations. The relationship between fast and slow gamma in experimental results were also plotted in Figure 8 (red and orange dots in Figure 8). In the parameter space of frequency change for the network without FB, the actual relationship between fast and slow gamma deviates from the simulated relationship a lot (Figure 8(c)). But the simulated relationship between fast and slow gamma covers the actual relationship in experiments when FB was added in the network (Figure 8(d)). In summary, the simulation results without FB could not satisfy the behavior of real data (the suppression index and frequency change for fast and slow gamma oscillation). However, the simulation results with FB could provide a possible solution for the behavior of experimental results.

## 4. Discussion

**Figure 9:**
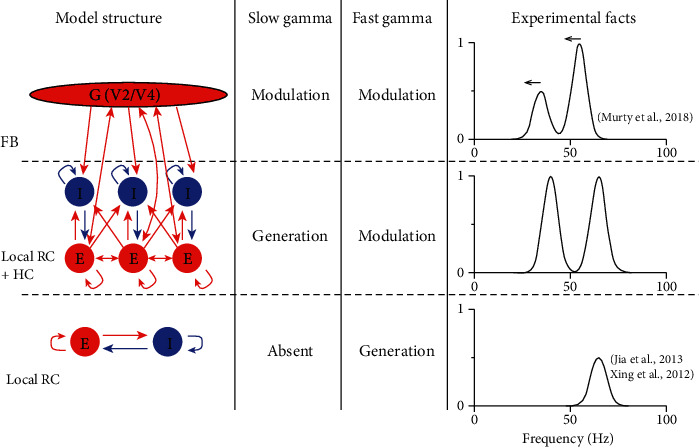
The summary for general roles of RC, HC, and FB in gamma oscillations in V1. This figure summarizes the roles of RC, HC, and FB for the generation and modulation of slow and fast gamma oscillations. Three rows in the figure show the function of local RC, HC, and FB to generate or modulate gamma oscillations. The demo of the power spectrum is shown in the right column.

This paper is the first study on the relationship between horizontal connection (HC) and gamma oscillation in a constructed large-scale model for monkey V1. Our work points out the function of HC for generating a new gamma oscillation that is different from the one generated by local RC. More importantly, we also found that HC and FB, the two types of large-scale connection patterns, have distinct modulatory effects on the response properties of gamma oscillations (Figure 9). Our results provided new insights into how gamma-band activity reflects different types of neural connection and give a theoretical prediction for distinguishing HC and FB effects based on the behaviors of gamma oscillations. Furthermore, the computational model, with multiple types of connection patterns, in this work not only reveals neural mechanisms for two distinct gamma oscillations in V1 of awake monkey but also can be used for studying multiple gamma oscillations in other brain regions.

### 4.1. Comparison with Previous Theoretical and Experimental Studies

It has been well studied for the generation of gamma oscillation in local circuits by mean-field [40, 53, 56, 66] or spiking neuron models [41–43, 67], and the modulation of gamma properties by FB connection was also well documented [4, 46]. Although some studies have built models with horizontal connection [30, 50] to understand V1 functions for information integration in visual space, to our best knowledge, the current study is the first to investigate the relationship between long-range horizontal connection and gamma oscillation. Model studies often created one global connection pattern and assumed this global connection included both FB and HC. However, our work indicates that FB and HC have to be modeled separately, because FB and HC have very different functional roles for gamma oscillations.

Our model could generate two distinct narrowband gamma oscillations in V1, which is highly consistent with a recent experimental work on awaked monkey [65]. However, several earlier studies only found one narrowband gamma oscillation in primary visual cortex experimentally [5, 12, 19, 68–71] or computationally [4, 40–43, 56, 67]. The reason for not finding prominent slow gamma oscillations in these studies might be due to different states of anesthesia [4, 56, 68, 72] or due to a smaller stimulus size [56, 65].

### 4.2. Different Roles of HC and FB

The anatomical connection for HC and FB is very different [24], but distinguishing and understanding the functional roles of HC and FB in the brain is still a challenging question [30]. The spike activity (neural firing rates) could be suppressed by large stimulus size through either HC or FB. It is hard for us to tell the different signatures for the two types of connection solely based on the response properties of spiking activity [14, 30, 38, 63, 73, 74]. However, our result clearly shows that gamma oscillations in networks with only HC are very different from those in networks with only FB: the slow gamma oscillation (Figure 3) is a signature of strong HC from Global E component to the local I component, but the modulation of slow gamma and fast gamma is a consequence of FB (Figure 4). This suggests that the response properties of gamma oscillations are essential measurements to distinguish HC and FB.

Gamma oscillation has been thought to play a role in information integration [1, 7–9]. But how to distinguish functional roles for two gamma peaks becomes a new challenging question. Some visual information might be involved with one gamma peak and others might be related to the other gamma peak. Because the generation of slow gamma requires a neural connection in a larger spatial scale and it could be modulated by FB connection, we speculate that slow gamma is related to information integration in a larger cortical/visual space.

### 4.3. Limitation of the Current Study

In this study, we have provided a large-scale neural network with many units modeled by the mean-field method. The advantage of this method is fast computation and easy to capture neural connections at multiple cortical scales. However, we do admit that the mean-field model does not describe local connection precisely, and it does not generate spike activity for individual neurons. Instead of spiking neuron model, it is an important tool more appropriate for modeling local circuitry and its detailed relationship to global connectivity. In order to further understand the neural mechanism of gamma oscillations, it will be ideal for applying a spiking neural network in our studies in the future.

Although we built a model with FB (connections from the high lever visual cortex such as V2 or V4) separated from HC, the global G component is a simplified version for the real high-level visual cortex. Connection pattern for FB and configuration for high-level visual cortex (G) in this work are based on the well-accepted concept as well as existing model settings in early studies [4, 46]. In reality, within higher level visual cortex, there are interactions between excitatory and inhibitory neurons as well [40], which is in a way similar to V1. In our opinion, it is reasonable to keep higher visual cortex as a single component G, since the effect of the feedback in our model is consistent with the model that has excitatory and inhibitory components in high-level brain regions [40]. Furthermore, the interaction of excitatory and inhibitory components in recurrent connection of higher level visual cortex remains unclear. The model for the higher visual cortex is another challenging question. We need more experimental results on the connection weights in high-level visual cortex to guide the modeling for the higher visual cortex.

## Figures and Tables

**Table 1:** Parameters in the model. Row 1 shows the time constants *τ*s. Rows 2-7 illustrates the values of coupling strength in the network.

	E_RC_	I_RC_	G
*τ* (seconds)	0.006	0.012	0.019
Coupling from LGN to	1.75	1.25	N/A
Coupling from E_RC_ to	1.5	3.5	0.1
Coupling from I_RC_ to	-3.25	-2.5	N/A
Coupling from E_HC_ to	0~0.03	0~5	N/A
Coupling from I_HC_ to	N/A	N/A	N/A
Coupling from G to	0~0.3	0~0.5	N/A

N/A: not applicable.

## Data Availability

The data of model simulation used to support the findings of this study are available from the corresponding author upon request.
